# The Convalescence of Dysentery and Its Complications

**Published:** 1918-05

**Authors:** 


**The Convalescence of Dysentery and its Complications.** By ALFRED HOWELL, Major R. A. M. C. (T). Abs. from *The Practitioner*, May, 1917.

The writer reviews his clinical notes of 200 convalescent cases of dysentery from Gallipoli. Histories of onset showed a remarkable number of men who had suffered from diarrhea, with or without passage of blood, but who did not " go sick " till a second attack, with passage of blood, so weakened the patient that he could no longer get about.

The subsequent complications observed were as follows : 13 cases of definite anemia; three cases of peripheral neuritis (serum examination in the two severer ones showed both Shiga and Flexner bacilli); two cases of arthritis; 16 of rheumatism; 2 of pneumonia (with severe diarrhea, checked by emetine, during the fever); two cases of enteric; and three of paratyphoid (two A and one B).

The peritonitis, perforation, and severe hemorrhage of acute dysentery were not seen.

